# Disentangling disorder-specific variation is key for precision psychiatry in autism

**DOI:** 10.3389/fnbeh.2023.1121017

**Published:** 2023-03-21

**Authors:** Aidas Aglinskas, Emily Schwartz, Stefano Anzellotti

**Affiliations:** Department of Psychology and Neuroscience, Boston College, Chestnut Hill, MA, United States

**Keywords:** individual differences, ASD, neuroimaging, machine learning, deep learning—artificial neural network, big data

## Introduction

Autism Spectrum Disorder (ASD) is highly heterogeneous across individuals, making it difficult to accurately diagnose and effectively treat each case. Genetics (Moreno-De-Luca and Martin, 2021), environment (Karimi et al., 2017), and intermediate brain phenotypes (Benkarim et al., 2021; Aglinskas et al., 2022) all contribute to this heterogeneity. In addition, the interplay between these factors is largely unknown. As a consequence, the identification of ASD subtypes and their causes remains challenging, hindering personalized diagnosis and treatment.

To complicate things, ASD-specific individual variation is entangled with variation that also occurs among neurotypical participants: ASD-related and unrelated factors jointly shape brain anatomy and function (Aglinskas et al., 2022). Furthermore, differences between data-acquisition sites and measurement errors additionally contribute to variation in the data collected for different individuals (Littmann et al., 2006). This is a challenge in multiple ways: at best, it reduces the effect sizes of reliable ASD biomarkers; at worst, it can produce spurious differences leading to subtypes that do not replicate across studies—as has been demonstrated in the case of depression (Drysdale et al., 2017; Dinga et al., 2019).

Therefore, in order to understand individual variation that is specifically related to ASD, we need to disentangle it from shared variation. However, separating ASD-specific variation from shared variation is difficult, and even recent studies typically do not do this. As a result, neural variation of interest for a disorder is often conflated with variation in age, gender and scanning site (Easson et al., 2019).

## Disentangling ASD-specific variation

Previous studies have attempted to identify ASD-specific patterns in neural data, using either case-control matching or normative models. Case-control matching compares ASD individuals with typically developing (TD) participants of matching characteristics (commonly age, gender, IQ, and scanning site). These approaches work well in theory, but assume that shared sources of variation are few and known (because participants must be selected taking these sources of variation into account). However, brain anatomy is shaped by a multitude of genetic and environmental factors (Gu and Kanai, 2014) some of which are unknown, undermining any attempt at explicit matching.

Normative models rely on pooling data from many TD and ASD participants and comparing their distributions to identify ASD-specific trends or developmental trajectories (Bedford et al., 2020). Such approaches can take into account sources of variation that are not explicitly matched, as long as the subject pool is diverse enough (Bethlehem et al., 2022). However, by design, normative models are models of groups, and they are not well suited for studying individual variation.

**Figure 1:**
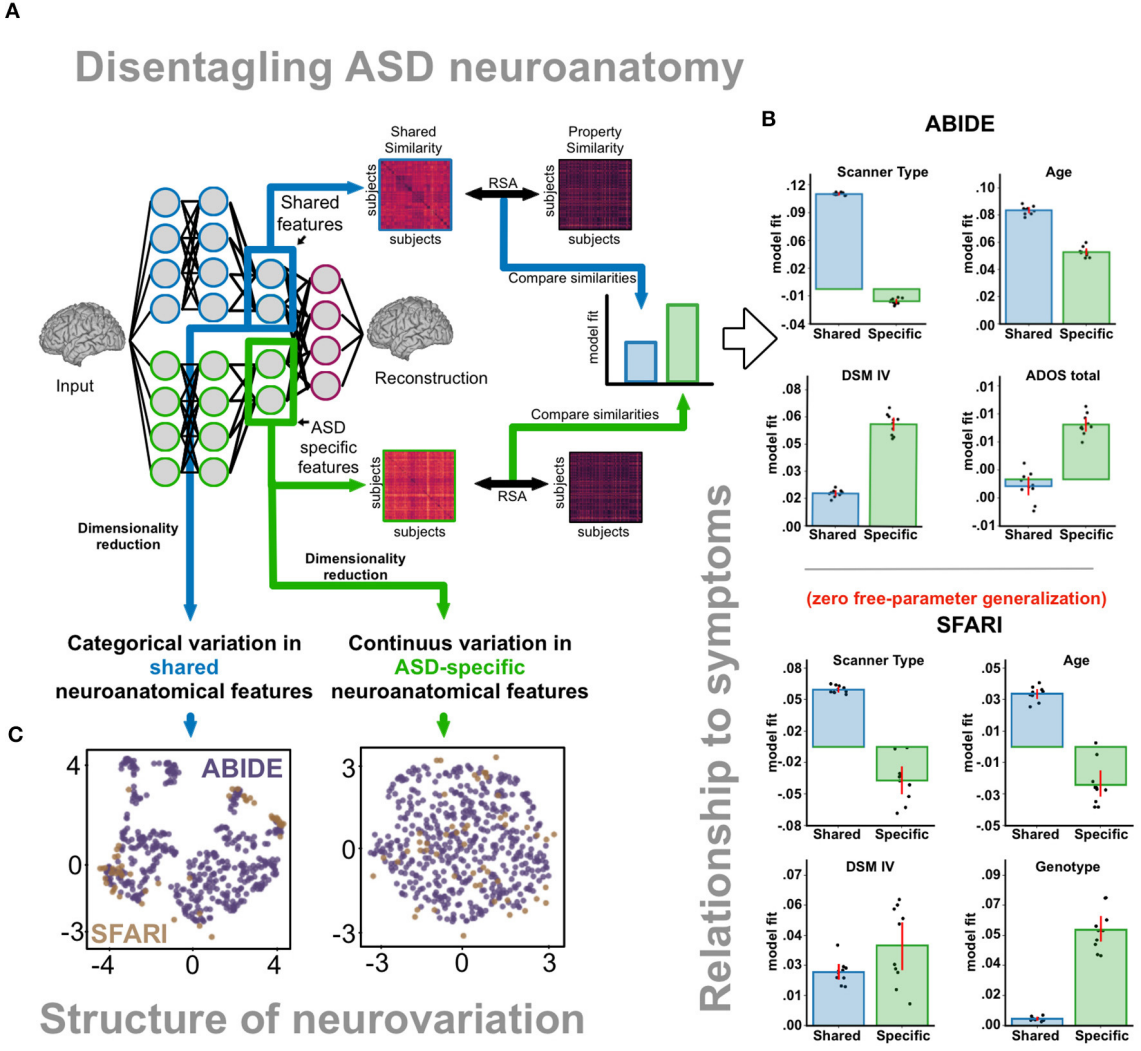
**(A)** Disentangling ASD neuroanatomy. After training the CVAE, we computed subject-specific similarity based on shared and ASD-specific neuroanatomical features for 512 ASD subjects. Shared and ASD-specific similarity matrices were then compared with expected similarities based clinical and non-clinical properties. **(B)** Relationship to symptoms. *ABIDE dataset:* ASD-specific features better correlated with properties indexing ASD severity (ADOS total, DSM IV), while shared features better correlated with non-clinical properties such as scanner type and age. Baseline model (non-contrastive variational autoencoder, which did not disentangle ASD-specific variation) features demonstrated worse relationships with both clinical and non-clinical properties. *SFARI dataset:* We replicated our findings using a zero-free-parameter generalization to an independent SFARI dataset with genotyped subjects. Neuroanatomical differences between 16p11.2 copy-number-variation subjects were better reflected in ASD-specific than shared features. **(C)** Structure of neurovariation. Dimensionality reduction (UMAP) revealed continuous rather than categorical variation in ASD-specific features. Categorical variation can instead be seen in shared features. B, Baseline model; Sh, Shared features; Sp, ASD-specific features.

To overcome the limitations of current approaches, it would be desirable to disentangle subject-specific ASD-specific features without the need of explicit matching (as in normative models), but at the level of individual participants (as in case-control matching). Contrastive variational autoencoders (CVAEs; Abid and Zou, 2019) are deep-learning models that can be trained to accomplish this, isolating ASD-specific features from features that are shared between ASDs and TDs (Figure 1A), including noise-driven variation that is observed in both the ASD and TD groups.

Recently, we applied these models to a large database of neuroanatomical scans (ABIDE I; Di Martino et al., 2014). We found that CVAEs improved over alternative methods in several key ways. First, CVAEs learned ASD-specific features that better reveal relationships between neuroanatomy and ASD symptoms (compared to approaches that do not disentangle ASD-specific variation, Figure 1B). Second, ASD-specific features generalized to an independent dataset (SFARI VIP), where we additionally demonstrated that ASD-specific, but not shared features, capture genotype variation carrying an increased risk of developing ASD (16p11.2 deletion and duplication). Third, ASD-specific features enable the identification of neuroanatomical loci implicated in ASD heterogeneity.

Finally, we observed that ASD-specific features are better described by continuous dimensions than categorical clusters (Figure 1C) suggesting that neuroanatomy is affected by ASD along smoothly varying factors, rather than distinct subtypes. This observation was only evident after disentanglement, as shared features still exhibited a clustered structure. Looking at ASD-specific variation can therefore more accurately represent how many distinct forms of Autism there are—potentially informing both nosology and treatment selection.

### The way forward

Even though recent advances show promise in tackling heterogeneity in ASD, more work remains to be done. For example, while the relationships between ASD symptoms and neuroanatomy are enhanced after disentangling, effect sizes remain small, indicating that a large portion of individual variation is not yet explained. This could be due to multiple reasons: coarse behavioral measures, relevant ASD-specific variation not being reflected in neuroanatomical data, or inability of the CVAEs to capture all the relevant information. Each of these represents an area of potential improvement.

### Improving behavioral measures

Current measures (such as ADOS, ADI-R, and Vineland scores) are designed for diagnosis using single aggregate scores; finer-grained measures might be needed to capture the multidimensional nature of ASD (Tang et al., 2020). Current measures do contain subdomains that can be used to capture some multidimensional variation (e.g., repetitive behavior or social-communication subdomains in ADOS). However, such experimenter-defined subdomains might not correspond to the dimensions of individual variation within ASD: a single dimension of individual variation might drive correlated changes across multiple subdomains, and vice versa. Therefore identifying dimensions that capture the difference between ASD participants and controls might not be sufficient to characterize all relevant individual variation within ASD. In addition, recent research has developed new computational approaches to measure social cognitive abilities such as the attribution of beliefs, desires, emotions, and traits (Baker et al., 2017; Houlihan et al., 2022). These approaches make it possible to estimate parameters that capture individual differences (“computational phenotyping”; Patzelt et al., 2018; Hauser et al., 2022). The convergence of computational phenotyping with techniques that disentangle disorder-specific variation such as CVAEs has the potential to paint a more detailed picture of individual differences in behavior associated with ASD.

### Integrating multiple data modalities

Analyzing jointly multiple kinds of data within each individual can offer a more complete understanding of individual variation. Data acquired with one modality can reveal features that are not visible in the data acquired with other modalities. For example, structural MRI can offer anatomical information that cannot be detected at the lower spatial resolution of fMRI; conversely, fMRI can reveal differences in functional responses that complement anatomical information. Furthermore, the joint analysis of different data modalities can reveal relationships between them. A recent study demonstrated this by examining jointly structural and functional MRI data (Hong et al., 2018). Structural MRI data identified 4 different subtypes of autism, and fMRI revealed different connectivity anomalies associated with each subtype (Hong et al., 2018). Similar strategies can be adopted to integrate other data modalities (e.g., EEG, MEG, and fMRI, see Mash et al., 2018).

Genetics can further complement the characterization of each participant. Joint analysis of genetic and neuroimaging data is of particular interest because it can reveal associations between genotypes and the resulting neurophenotypes. Moving an initial step in this direction, a recent study used fMRI to measure alterations in functional connectivity in ASD, and compared their location to gene expression maps for genes associated with ASD (Benkarim et al., 2021). Another recent study (Lu et al., 2022) found that combining genetics and fMRI data improved ASD classification, suggesting that these data modalities offer complementary information.

Future studies will need to leverage large datasets to link genetics, neurophenotypes and symptoms. In particular, acquiring neuroimaging and behavioral measures as part of newly collected genetic datasets will be essential to understand the impact of individual differences in genetics on the brain and on behavior. In this context, disentangling ASD-specific variation from shared variation in neural and behavioral measures will help guide the identification of genes associated with ASD phenotype.

### Addressing comorbidity

ASD rarely occurs in isolation—ADHD and anxiety are just some of the most frequently co-occurring conditions (Matson and Goldin, 2013). Individuals with comorbid ASD consistently show higher severity scores (Hayashi et al., 2022), making it difficult to discern based on the ADOS scores alone whether a person has severe or comorbid autism. Extremely high rates of comorbidity (ADHD is present in 50–70 percent of ASD individuals) suggest that perhaps comorbidity might be better conceptualized as another facet of individual variation.

It is therefore pressing to understand the neural bases of comorbidity in order to inform the etiology of ASD. For example: Do individuals with comorbid ASD+ADHD present with a conjunction of neural features seen in individuals who have only ASD and only ADHD, or with a distinct set of features? Research has identified both distinct and overlapping features in comorbid ADHD+ASD but definitive etiology of this condition remains elusive (Hours et al., 2022).

Several other neurological conditions (epilepsy, early onset stroke, Parkinsons; Brainstorm Consortium et al., 2018), gastrointestinal issues (Martínez-González and Andreo-Martínez, 2019), and psychiatric disorders (Obsessive Compulsive Disorder, Schizophrenia, Bipolar disorder, and Major Depressive Disorder) are comorbid with ASD (Brainstorm Consortium et al., 2018). Recent studies are revealing shared genetic, neural and cognitive mechanisms between ASD and comorbid psychiatric disorders (Mizuno et al., 2019; Thorp et al., 2021). CVAEs—with appropriate architecture modifications—can help to identify features specific to the presence of each disorder in isolation as well as features that are uniquely associated with comorbidity.

### Leveraging state of the art techniques

There are already a variety of computational approaches in psychiatry (see Graham et al., 2019). State-of-the-art deep learning methods have been used to distinguish between ASD participants and controls (Alsaade and Alzahrani, 2022; Chen et al., 2022; Santana et al., 2022), but are just beginning to be applied to investigate ASD subtypes and factors underlying ASD variation (Akter et al., 2021; Aglinskas et al., 2022; Parlett-Pelleriti et al., 2022). In studying individual variation specifically, even the methods that have been applied have not been fully tested in all their possible variants. For example, in deep networks, the dimensionality of the layers and their number are tied to model performance. However, larger models are prone to overfitting, therefore training set size, and diversity become increasingly important (Dinsdale et al., 2022), as well as the use of regularization techniques (e.g., dropout). Increasing model size in CVAEs while taking adequate steps to mitigate overfitting could better capture ASD-specific variation.

Alternatively, breakthroughs could come from utilizing novel network architectures. For example, Generative Adversarial Networks and Diffusion models (Goodfellow et al., 2020; Rombach et al., 2021) are able generate more realistic outputs than VAEs (on which CVAE is based on), often indistinguishable from real inputs (Karras et al., 2018; Yi et al., 2019). This ability could prove advantageous if it captures finer-grained information that might have been lost in CVAEs.

A challenge for these models remains making latent dimensions explicit (conditioning on features like age, diagnosis, or genotype) or at least interpretable. A recent study went in this direction, predicting disease-specific brain aging without longitudinal data conditioning generative models using disorder and age information (Xia et al., 2019). Applied to the case of ASD, these approaches might be used to predict individuals' response to treatment (conditioning on current neuroimaging data) without the need for traditional factorial design, i.e., all participants completing all treatments. Disentangling ASD-specific variation would make it possible to condition the models selectively using neuroimaging features that are relevant to ASD, removing confounding features that could impair the models' performance.

### Linking descriptive research with intervention studies

Descriptive research into ASD biomarkers and subtypes must go hand in hand with intervention studies aimed at improving the quality of life in affected individuals. While there are various non-medical interventions available for ASD treatment (Bond et al., 2016), response across individuals is highly variable (Kamio et al., 2015). ASD individuals respond to some interventions, but not others (Zachor and Ben-Itzchak, 2017), suggesting that selecting the right treatment for the right individual is critical. Currently, targeting specific treatments based on behavioral profiles shows only limited success in predicting response to treatment and ASD progression (Hollocks et al., 2022). Recently, fMRI biomarkers have shown promise in predicting response to treatment better than behavioral measurements (Yang et al., 2016)—disentangling disorder-specific variation can potentially bring such neuromarker approaches closer to clinical validity. In order to bridge the gap between descriptive studies and the development of personalized interventions, research findings should increasingly be used to predict future symptom trajectories and the interventions that individuals are most likely to respond to Bzdok et al. (2021).

## Conclusions

Individual differences are a key challenge in understanding biological bases of ASD and developing targeted treatments. The field can tackle this challenge by making concerted progress along several key research directions: improving the behavioral characterization of participants, integrating data modalities, modeling comorbidity, taking advantage of new computational techniques, and ultimately studying the link between individual variation and intervention outcomes. Across all these research directions, disentangling disorder-specific variation from unrelated variation will be an essential asset.

## Author contributions

AA and ES: conceptualization, research, and writing. SA: conceptualization, research, writing, funding acquisition, and supervision. All authors contributed to the article and approved the submitted version.
